# Bibliometric analysis of research on the role of intestinal microbiota in obesity

**DOI:** 10.7717/peerj.5091

**Published:** 2018-06-29

**Authors:** Haiqiang Yao, Jin-Yi Wan, Chong-Zhi Wang, Lingru Li, Ji Wang, Yingshuai Li, Wei-Hua Huang, Jinxiang Zeng, Qi Wang, Chun-Su Yuan

**Affiliations:** 1 Beijing University of Chinese Medicine, Beijing, China; 2 Tang Center for Herbal Medicine Research, The University of Chicago, Chicago, IL, United States of America; 3 Department of Anesthesia and Critical Care, The University of Chicago, Chicago, IL, United States of America; 4 School of Pharmacy, Jiangsu University, Zhenjiang, China

**Keywords:** Bibliometrics, Obesity, Intestinal microbiota, Data-mining

## Abstract

**Background:**

Obesity is a key public health problem. The advancement of gut microbiota research sheds new light on this field. This article aims to present the research trends in global intestinal microbiota studies within the domain of obesity research.

**Methods:**

Bibliographic information of the publications on intestinal microbiota and obesity was retrieved from the Scopus database, and then analyzed by using bibliometric approaches.

**Results:**

A total of 3,446 references were retrieved; the data indicated a steady growth and an exponential increase in publication numbers. The references were written in 23 different languages (93.8% in English). A number of 3,056 English journal papers were included in the further analyses. Among the 940 journals, the most prolific ones were *PLOS ONE*, *Scientific Reports,* and *British Journal of Nutrition*. North America and Europe were the highest publication output areas. The US (995 publications) ranked first in the number of publications, followed by the China (243 publications) and France (242 publications). The publication numbers were significantly correlated with gross domestic product (GDP), human development index (HDI), and population number (PN). International collaboration analysis also shows that most of the collaborations are among developed countries.

**Discussion:**

This comprehensive bibliometric study indicates that gut microbiota is a significant topic in the obesity research. The structured information may be helpful in understanding research trends, and locating research hot spots and gaps in this domain.

## Introduction

Obesity, marked by the excess body fat accumulation, is a major public health crisis. This medical condition has almost become a global epidemic and is progressing rapidly (Barbieri et al., 2017; D’Souza et al., 2017). Among factors, intestinal microbiota can influence the whole-body metabolism by affecting the host’s energy homeostasis (De Vadder & Mithieux, 2018; Omari-Siaw et al., 2016). There are ample studies showing that probiotics and prebiotics, or other dietary substances, can be used to alleviate obesity through the modulation of the host’s intestinal microbiota (Bird et al., 2017; Li et al., 2017a; Li et al., 2017b). However, to date, the role of intestinal microbiota in the formation and progression of obesity has not been systematically presented using bibliometric analysis.

Bibliometric analysis can be applied to track the developing trends, access the influences of publications, and compare the academic performance between different regions of a certain research field. This bibliometric technique has been applied in the domain of obesity; one publication investigated studies from 1998–2007 using data from PubMed (Vioque et al., 2010), and another study analyzed the longitudinal trends from 1993 to 2012 with Scopus as the data source (Khan et al., 2016). However, to our knowledge thus far, no bibliometric studies have assessed intestinal microbiota and obesity research at the global level.

The aim of this study was to present an overview of the research trends on intestinal microbiota and obesity up to now and shed new light on future research directions. We investigated the growth and citation of publications, active authors, countries and institutions, international collaboration, and the frequency of terms through a bibliometric analysis.

## Methods

### Search strategy

Bibliometric data can be acquired through various search engines. In this study, the Scopus database was selected to perform the literature search for all published articles on enteric microbiota and obesity. It was justifiable to use Scopus as our data source to retrieve abstracts, citations and other bibliometric data given that it has wider resources and is consistently more accurate than other alternatives such as PubMed, Web of Science and Google Scholar (Choudhri et al., 2015; Falagas et al., 2008; Kulkarni et al., 2009).

The Scopus database was searched from its inception to December 31, 2017 with no language limitation. The synonyms for gut microbiota and obesity were included in the search strategy. The keywords pertaining to gut microbiota were: gastrointestinal microbiomes, gut microflora, gut microbiota, gastrointestinal flora, gut flora, gastrointestinal microbiota, gut microbiome, gastrointestinal microflora, intestinal microbiome, intestinal microbiota, intestinal microflora, and intestinal flora. The keywords regarding obesity were: obesity, corpulence, fatness, and overweight. The two sets of keywords were searched with the AND logic in the Article title/ Abstract/ Keywords fields. The final search query was built like this:

((TITLE-ABS-KEY ( gastrointestinal AND microbiomes ) OR TITLE-ABS-KEY ( gut AND microflora ) OR TITLE-ABS-KEY ( gut AND microbiota ) OR TITLE-ABS-KEY ( gastrointestinal AND flora ) OR TITLE-ABS-KEY ( gut AND flora ) OR TITLE-ABS-KEY ( gastrointestinal AND microbiota ) OR TITLE-ABS-KEY ( gut AND microbiome ) OR TITLE-ABS-KEY ( gastrointestinal AND microflora ) OR TITLE-ABS-KEY ( intestinal AND microbiome ) OR TITLE-ABS-KEY ( intestinal AND microbiota ) OR TITLE-ABS-KEY ( intestinal AND microflora ) OR TITLE-ABS-KEY ( intestinal AND flora ) ) AND PUBYEAR <2018 ) AND ( ( TITLE-ABS-KEY ( obesity ) OR TITLE-ABS-KEY ( corpulence ) OR TITLE-ABS-KEY ( fatness ) OR TITLE-ABS-KEY ( overweight ) ) AND PUBYEAR <2018 ).

All of the citation information, bibliographical information, abstract and keywords, funding details and other information of the retrieved publications was exported with CSV format through the University of Chicago Library’s access, for the further data processing.

### Data analysis

All bibliometric information was exported into CSV format from the Scopus database. Microsoft Excel was applied to for sorting and to perform statistical procedures. Top prolific authors, countries, journals, institutions and most cited papers were ranked according to the standard competition ranking (SCR). Data visualization was conducted using the VOSviewer technique to create scientific landscapes and networks based on the citation frequency, countries, journals, authors and other information (Van Eck & Waltman, 2010). GunnMap 2 (http://lert.co.nz/map/) was used to generate the world map to show the publication distribution.

Some bibliometric indicators were applied in the analysis. The strength of publications included was assessed by impact factor (IF) obtained from the latest Journal Citation Report (2016) published by Thomson Reuters. Bradford’s Law was used as a scattering index to reveal the distribution of the scientific literature in a particular discipline. Bradford proposed a model of concentric zones of productivity, termed as Bradford zones, with decreasing densities of literature. Each of these zones would contain a similar number of articles, but the number of journals in which these articles are published would increase on passing from one zone to another (Bradford, 1948). This model permits the identification of journals that are most widely used or have the greatest weightage in a given field of scientific production.

GraphPad Prism version 6.0c (San Diego, CA, USA) was used to conduct statistical analysis. Pearson’s correlation analysis was used to investigate relationships between the publication numbers of different countries/regions and some related variables, such as gross domestic product (GDP), GDP per capita (purchasing power parity, PPP), etc. A *P* value of <0.05 was considered statistically significant.

## Results

### Publications analysis based on numbers and citations

**Figure 1:**
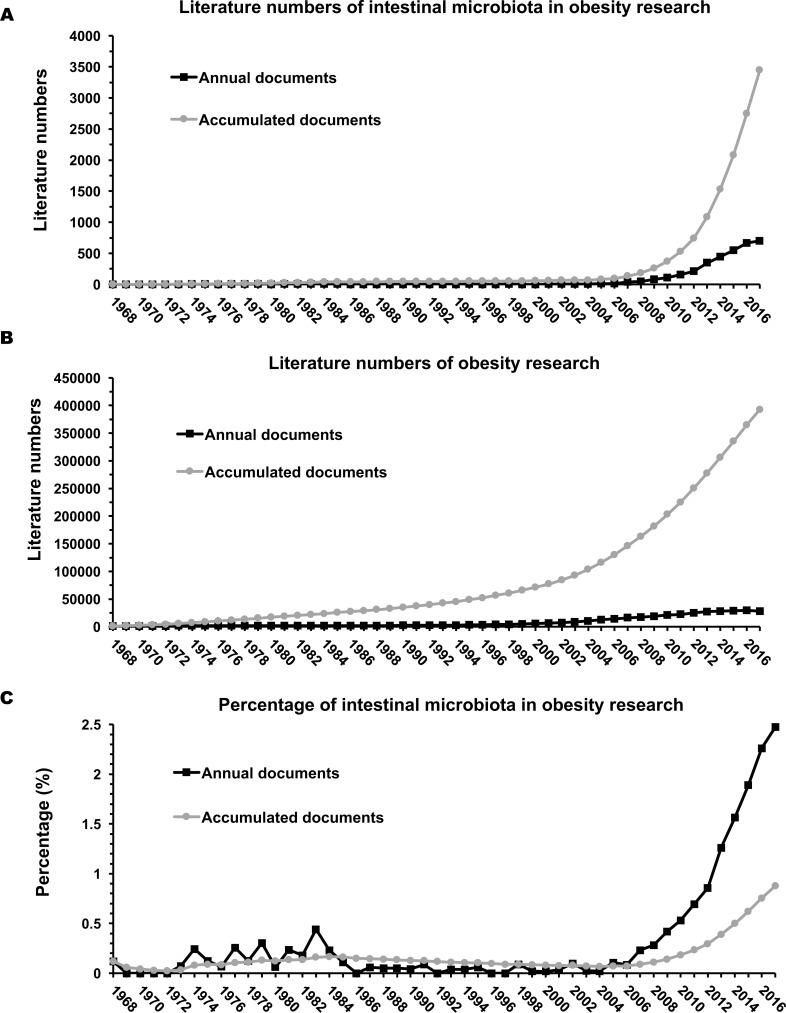
Annual and accumulated publications of intestinal microbiota and obesity (A), obesity research (B), and the percentage of intestinal microbiota related publications in the obesity research (C).

The applied strategy yielded a total of 3,446 publications. The first article on gut microbiota and obesity was published in 1968, and the annual publication numbers were stable in the following nearly 40 years till 2004. A dramatic growth was observed in the last 15 years from 2003 to 2017, and the number of annual documents during this period showed an exponential growth trend (*y* = 0*e*^0.4263*x*^, *R*^2^ = 0.94). The specific numbers of annual documents and accumulated documents are shown in Fig. 1A. The highest number of annual publications was seen in 2017, totaling 702 publications. The publication numbers of obesity research (Fig. 1B) can serve as a background frame to present a better understanding of the growth trend of intestinal microbiota in obesity research. As shown in Fig. 1C, the percentage of intestinal microbiota related publications in the obesity research was increasing gradually, especially after the year of 2006.

**Figure 2:**
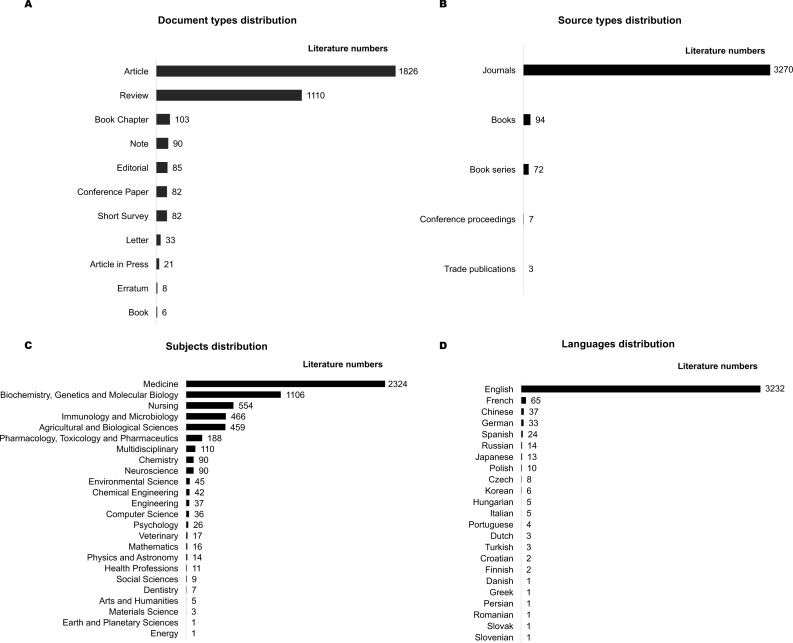
General information regarding the 3,446 publications retrieved on gut microbiota and obesity. (A) Document types distribution; (B) Source types distribution; (C) Subjects distribution; (D) Languages distribution.

In terms of document type, the majority of the retrieved papers were research articles (*n* = 1826, 53.0%), followed by review articles (*n* = 1110, 32.2%) and book chapters (*n* = 103, 3.0%) (Fig. 2A). The primary source of the publications was journals (Fig. 2B). As shown in Fig. 2C, most of the retrieved documents belong to medical subjects (*n* = 2, 324, 67.4%), followed by Biochemistry, Genetics and Molecular Biology (*n* = 1, 106, 32.1%), Nursing (*n* = 554, 16.1%), Immunology and Microbiology (*n* = 466, 13.5%), and Agricultural and Biological Sciences (*n* = 459, 13.3%). The retrieved papers were written in 23 different languages, mainly in English (*n* = 3232, 93.8%), followed by French (*n* = 65, 1.9%), Chinese (*n* = 37, 1.1%), German (*n* = 33, 1.0%), and Spanish (*n* = 24, 0.7%).

All of the retrieved 3,446 papers were cited a total of 141,918 times. A total of 2,858 (82.9%) articles had at least one citation while 588 (17.1%) articles had no citations.

Among the total 3,446 documents there were multiple source types (Fig. 2B) that were written in different languages (Fig. 2D). Considering the heterogeneity of the documents may add background noises to the study, we only included the journal articles written in English to perform the further analyses. According to this inclusion criteria, a total of 390 documents (books, book series, conference proceedings and other types) were excluded. 3,056 English papers were included for the subsequent analyses.

### Publications analysis based on top cited articles

Top 20 cited papers are listed in Table 1 (Bäckhed et al., 2004; Bäckhed et al., 2005; Bäckhed et al., 2007; Cani et al., 2007; Cani et al., 2008; Cani et al., 2009; Henao-Mejia et al., 2012; Kau et al., 2011; Le Chatelier et al., 2013; Ley et al., 2005; Ley et al., 2006; O’Hara & Shanahan, 2006; Qin et al., 2010; Ridaura et al., 2013; Tremaroli & Bäckhed, 2012; Turnbaugh et al., 2008; Turnbaugh et al., 2009a; Turnbaugh et al., 2006; Turnbaugh et al., 2009b; Vijay-Kumar et al., 2010). The highest citation number was 3,961 for the article entitled “An obesity-associated gut microbiome with increased capacity for energy harvest” (Turnbaugh et al., 2006). Among the top 20 highly cited papers, eight were published in *Nature*, followed by *Science* and *Proceedings of the National Academy of Sciences of the United States of America* (three papers), *Diabetes* (two papers). As the first author, Turnbaugh PJ published four papers among the top 20 cited papers, followed by Bäckhed F. and Cani P.D. (three papers), then by Ley RE (two papers). Among the top 20 cited papers, Gordon, J.I. participated 12 papers, followed by Ley R.E. (six papers) and Bäckhed F. (five papers); they were the most productive of the highest quality authors.

**Table 1:** Top 20 cited articles on gut microbiota and obesity from inception to 2017.

SCR	Article	Title	Year	Source title	Cited by	IF
1st	Turnbaugh et al. (2006)	An obesity-associated gut microbiome with increased capacity for energy harvest	2006	Nature	3,961	40.137
2nd	Qin et al. (2010)	A human gut microbial gene catalogue established by metagenomic sequencing	2010	Nature	3,724	40.137
3rd	Turnbaugh et al. (2009a)	A core gut microbiome in obese and lean twins	2009	Nature	3,072	40.137
4th	Ley et al. (2006)	Microbial ecology: Human gut microbes associated with obesity	2006	Nature	3,023	40.137
5th	Bäckhed et al. (2004)	The gut microbiota as an environmental factor that regulates fat storage	2004	PNAS	2,350	9.661
6th	Ley et al. (2005)	Obesity alters gut microbial ecology	2005	PNAS	2,292	9.661
7th	Bäckhed et al. (2005)	Host-bacterial mutualism in the human intestine	2005	Science	2,174	37.205
8th	Cani et al. (2007)	Metabolic endotoxemia initiates obesity and insulin resistance	2007	Diabetes	1,986	8.684
9th	Cani et al. (2008)	Changes in gut microbiota control metabolic endotoxemia-induced inflammation in high-fat diet-induced obesity and diabetes in mice	2008	Diabetes	1,521	8.684
10th	Tremaroli & Bäckhed (2012)	Functional interactions between the gut microbiota and host metabolism	2012	Nature	1,198	9.661
11st	Turnbaugh et al. (2009b)	The effect of diet on the human gut microbiome: a metagenomic analysis in humanized gnotobiotic mice	2009	Science Translational Medicine	1,091	16.761
12nd	Bäckhed et al. (2007)	Mechanisms underlying the resistance to diet-induced obesity in germ-free mice	2007	PNAS	1,081	9.661
13rd	Turnbaugh et al. (2008)	Diet-induced obesity is linked to marked but reversible alterations in the mouse distal gut microbiome	2008	Cell Host and Microbe	1,046	14.946
14th	Vijay-Kumar et al. (2010)	Metabolic syndrome and altered gut microbiota in mice lacking toll-like receptor 5	2010	Science	996	37.205
15th	O’Hara & Shanahan (2006)	The gut flora as a forgotten organ	2006	EMBO Reports	949	8.568
16th	Cani et al. (2009)	Changes in gut microbiota control inflammation in obese mice through a mechanism involving GLP-2-driven improvement of gut permeability	2009	Gut	903	16.658
17th	Le Chatelier et al. (2013)	Richness of human gut microbiome correlates with metabolic markers	2013	Nature	900	40.137
18th	Kau et al. (2011)	Human nutrition, the gut microbiome and the immune system	2011	Nature	885	40.137
19th	Henao-Mejia et al. (2012)	Inflammasome-mediated dysbiosis regulates progression of NAFLD and obesity	2012	Nature	884	40.137
20th	Ridaura et al. (2013)	Gut microbiota from twins discordant for obesity modulate metabolism in mice	2013	Science	882	37.205

**Notes.**

Abbreviation SCR standard competition ranking. Equal items were given the same ranking number, and then a gap is left in the ranking numbers PNAS Proceedings of the National Academy of Sciences of the United States of America IF impact factor

a Data extracted from Journal Citation Reports, Thomson Reuters, 2016.

### Publications analysis based on authors

A total of 10,648 authors contributed to the publication of all the 3,056 papers included. The number of authors for a single document, also known as the transience index, is 8,224, accounting for 77.2% of all the authors. There are 85 authors who published more than ten articles in this field. Cani, P.D. was the most productive author in this field with 76 publications, the following were Delzenne, N.M. (52 publications) and Bäckhed, F. (48 publications). Details of the top 20 most prolific authors are presented in Fig. S1A, and the co-authorship analysis is shown in Fig. S2.

**Figure 3:**
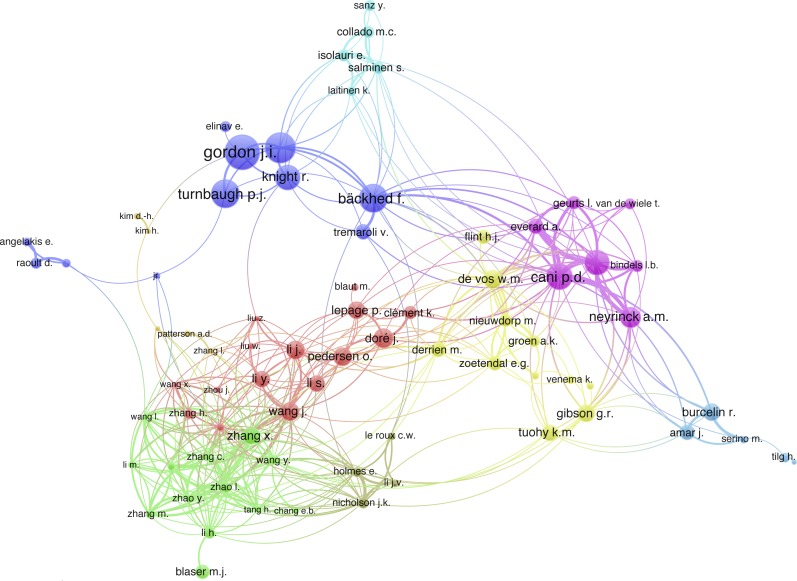
Citation analysis of 85 authors with at least 10 publications. Thicker lines indicate stronger collaborations. Authors represented with larger circle size or font size had relatively more citations.

A total of 9,590 authors have been cited at least once, accounting for 91.7% of the total 10,462 authors, 6,064 authors had citation numbers at least 10 (58.0%), and 1,597 authors have been cited at least 100 times (15.3%). Gordon JI was the most influential author with the greatest citation number of 25,073, followed by Ley R.E. (17,658), and Bäckhed F. (15,518); details of citation analysis are represented in Fig. 3. Among the top 20 most cited authors, Batoo, J-M, Levenez, F and Renault, P had the greatest citation numbers per publication (1,303.8), followed by Tap, J (1,297.5) and Parkhill, J (1,291.5), more details are shown in Fig. S1B.

### Publications analysis based on countries/regions

**Figure 4:**
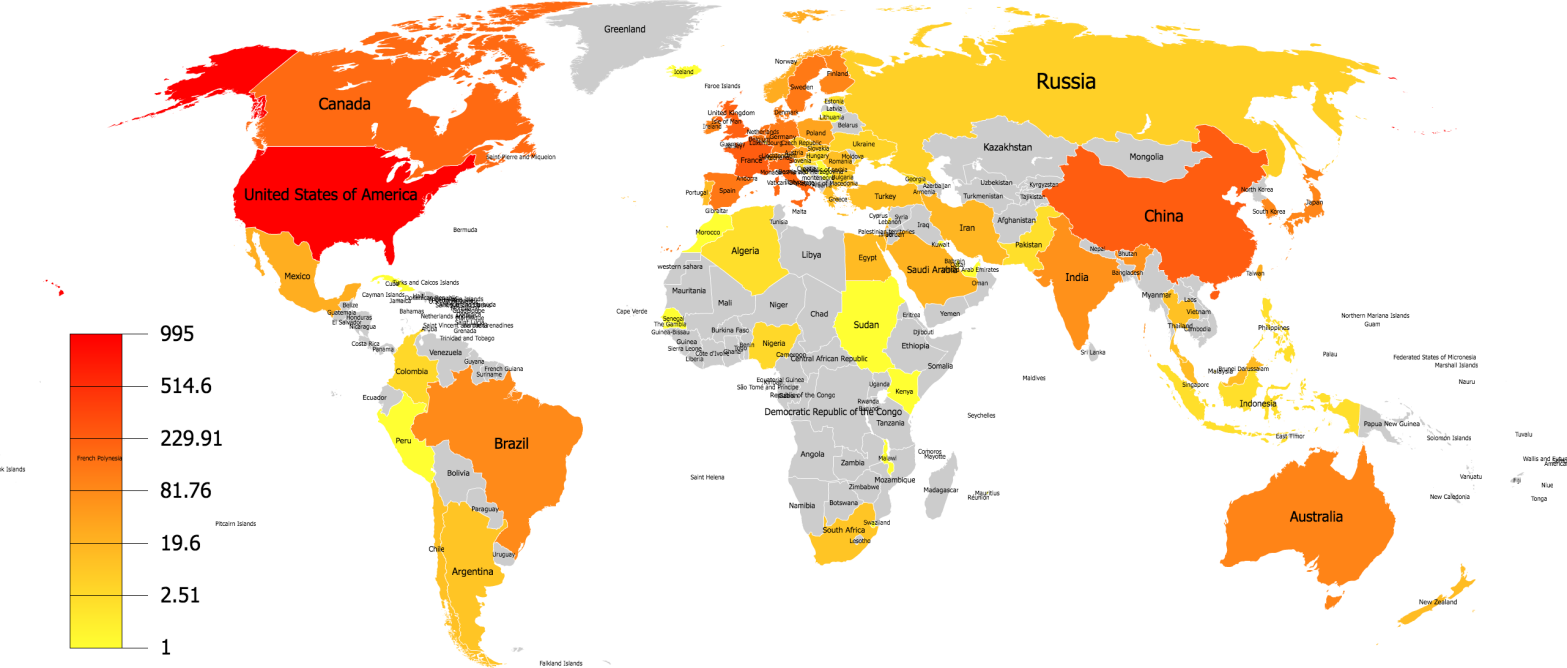
Geographical distribution map of publications on intestinal microbiota in obesity research.

The geographical distribution of publications involved 79 countries/regions over six continents (Fig. 4). There were 20 countries that just published only one article, and 46 countries that published at least five articles. The top 20 most productive countries are shown in Fig. S1C; the US ranked first with 995 publications, followed by China (243 publications), France (242 publications), United Kingdom (223 publications), and Italy (196 publications). However, the GDP-adjusted ranking according to their productivity scores was different, the most prolific countries were Finland, Denmark, Ireland, Belgium, and Swaziland (Fig. S1D). Publication productivity scores of countries/regions were calculated by dividing the number of publications by their GDP and then multiplying the result with by 1E + 12. As shown in the Fig. S1E, the most productive continents according to the publication numbers were Europe, North America, and Asia.

**Figure 5:**
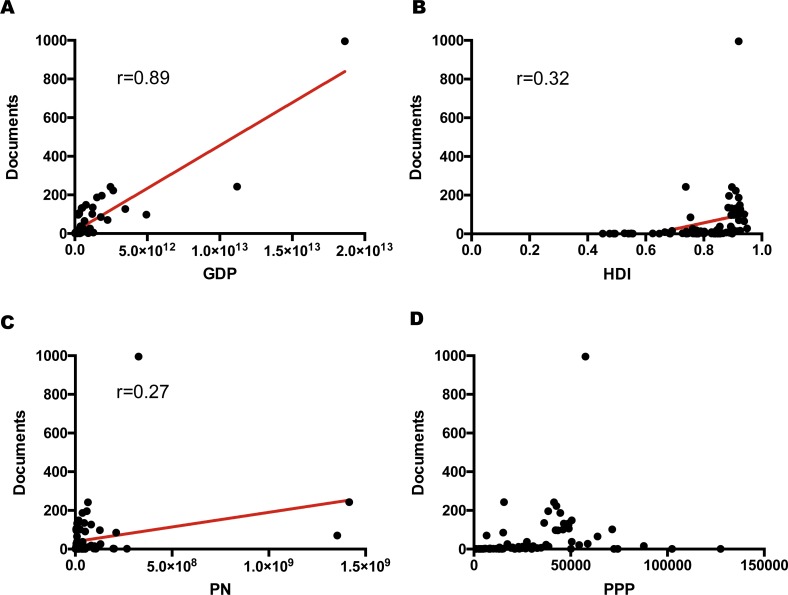
Correlations between the publication number of bariatric intestinal microbiota in obesity research and (A) GDP (*r* = 0.89, *p* < 0.0001), (B) HDI (*r* = 0.32, *p* < 0.001), (C) PN (*r* = 0.27, *p* < 0.05), and (D) PPP (*p* = 0.08). Abbreviations: GDP, gross domestic product; HDI, human development index; PN, population number; PPP, purchasing power parity (gross domestic product per capita).

In order to achieve a better understanding of the diverse publication numbers in different countries/regions, the relationship between the publication numbers and multiple factors for each country was investigated. The analyzed factors were population number (PN), gross domestic product (GDP), gross domestic product per capita (purchasing power parity, PPP), and human development index (HDI). Significant correlation was found between the publication number of intestinal microbiota in obesity research and GDP, HDI, and PN (Fig. 5). GDP demonstrated the highest correlation with the publication number (*r* = 0.89, *p* < 0.0001), followed by HDI (*r* = 0.32, *p* < 0.001) and PN (*r* = 0.27, *p* < 0.05). GDP was the most important factor that could benefit the publication productivity. Therefore, this analysis can interpret the reason why the US and China were the most prolific countries in this domain.

Analysis of citation counts for countries showed that the US had the most citations followed by France, UK, Belgium and the Netherlands (Fig. S3). The international collaboration analysis based on these countries is shown in a network visualization map (Fig. S4). As is indicated in the collaboration analysis, the United States (US) had collaborations with other 38 countries and followed by Italy (35 collaboration links), Spain (33 collaboration links), United Kingdom (UK) (32 collaboration links), and Germany (31 collaboration links). For the US, collaboration was mostly with China (relative link strength = 64) and Canada (relative link strength = 45).

### Publications analysis based on institutions

Prolific institutions in publishing papers on gut microbiota and obesity are presented in Table 2. The most active institution was *University College Cork* in Ireland (122 publications), followed by *The Institut national de la santé et de la recherche médicale* (Inserm) in France (109 publications), and *Universite Catholique de Louvain* in Belgium (95 publications). A total of 160 institutions published at least 10 articles. Among the top 20 most active institutions, 14 are in Europe, four and in North America, and two are in China. A total of 79 organizations have been cited at least 1,000 times, the citation analysis was shown in Fig. S5. *The Department of Chemistry and Biochemistry, University of Colorado* obtained the highest citation number (7,930 citations), and followed by *Center for Genome Sciences, Washington University in St. Louis* (5,032 citations) and *Danone Research*, in France (4,652 citations).

**Table 2:** Top 20 prolific institutions in publishing papers on gut microbiota and obesity.

SCR	Institution	Country	Documents	% *N* = 3, 056
1st	University College Cork	Ireland	122	4.0
2nd	Inserm	France	109	3.6
3rd	Universite Catholique de Louvain	Belgium	95	3.1
4th	Kobenhavns Universitet	Denmark	87	2.8
5th	Goteborgs Universitet	Sweden	74	2.4
6th	Wageningen University and Research Centre	The Netherlands	60	2.0
7th	INRA Institut National de La Recherche Agronomique	France	53	1.7
7th	The Wallenberg Laboratory	Sweden	53	1.7
9th	Imperial College London	UK	49	1.6
10th	VA Medical Center	US	46	1.5
11st	Harvard Medical School	US	45	1.5
11st	Helsingin Yliopisto	Finland	45	1.5
13rd	CNRS Centre National de la Recherche Scientifique	France	41	1.3
14th	Novo Nordisk Foundation	Denmark	40	1.3
14th	Consejo Superior de Investigaciones Científicas	Spain	40	1.3
16th	Chinese Academy of Sciences	China	39	1.3
16th	Shanghai Jiao Tong University	China	39	1.3
18th	University of Calgary	Canada	37	1.2
19th	Maastricht University	The Netherlands	36	1.2
20th	University of Chicago	US	33	1.1

### Publications analysis based on journals

**Figure 6:**
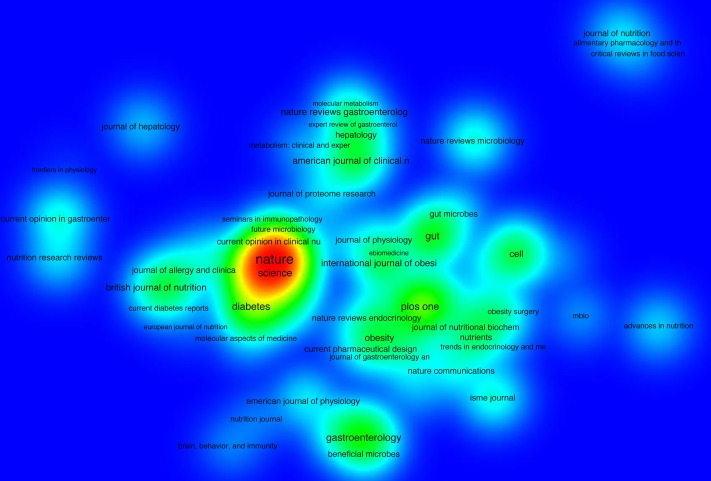
Density map of journals citation analysis. One hundred twenty-seven journals were included in this analysis with a minimum productivity of five publications in this field. Journals with the higher number of citations have darker spots.

All the retrieved documents were published in 940 different journals. The top 20 active journals in publishing articles on gut microbiota and obesity are shown in Table 3. The most prolific journal in this field was *PLOS ONE* (106 publications), followed by *Scientific Reports* (46 publications), and *British Journal of Nutrition* (43 publications). The total number of articles published in top 20 journals was 728, accounting for 23.8% of total retrieved documents. Citations analysis of the included 143 journals with at least five publications indicated that *Nature* has the highest citation numbers (*N* = 22, 139), followed by *Proceedings of The National Academy of Sciences of The United States of America* (*N* = 10, 195), and *PloS One* (*N* = 5, 117) (Fig. 6). Regarding the academic journals publishing articles on gut microbiota and obesity, the Bradford model was applied. The division of Bradford’s zones in this study is shown in Table 4. A total of 940 journals were involved; the average number of articles in each zone was 305.6. The first zone included six journals: *Plos One, Scientific Reports, British Journal of Nutrition, Nutrients, Gut Microbes,* and *Cell Metabolism.*

**Table 3:** Top 20 prolific journals in publishing papers on gut microbiota and obesity.

SCR	Journals	Documents	% *N* = 3, 056	IF
1st	Plos One	106	3.5	2.806
2nd	Scientific Reports	46	1.5	4.259
3rd	British Journal of Nutrition	43	1.4	3.706
4th	Nutrients	42	1.4	3.55
5th	Gut Microbes	40	1.3	NA
5th	Cell Metabolism	37	1.2	18.164
7th	Frontiers in Microbiology	36	1.2	4.076
7th	World Journal of Gastroenterology	36	1.2	3.365
9th	Gastroenterology	33	1.1	18.392
10th	Nature	32	1.0	40.137
11st	Beneficial Microbes	30	1.0	2.923
11st	Molecular Nutrition and Food Research	30	1.0	4.323
11st	Nature Reviews Gastroenterology and Hepatology	30	1.0	13.678
14th	Gut	29	0.9	16.658
14th	International Journal of Obesity	29	0.9	5.487
16th	American Journal of Clinical Nutrition	27	0.9	6.926
16th	Current Opinion in Clinical Nutrition and Metabolic Care	27	0.9	4.023
18th	Nature Reviews Endocrinology	26	0.9	18.318
19th	Journal of Nutrition	25	0.8	4.145
20th	Journal of Nutritional Biochemistry	24	0.8	4.518

**Notes.**

Abbreviation NA not available

**Table 4:** Distribution of the journals in Bradford’s zones.

Bradford’s Zones	Number of Journals	% Journals	Number of articles	Bradford’s multiplier
1	6	0.6	314	
2	10	1.1	312	1.67
3	15	1.6	304	1.50
4	24	2.6	300	1.60
5	35	3.7	302	1.46
6	53	5.6	305	1.51
7	88	9.4	305	1.66
8	139	14.8	304	1.58
9	265	28.2	305	1.91
10	305	32.4	305	1.15
Total number of journals = 940				
Average number of articles in each zone = 305.6				

### Publications analysis based on terms frequency

**Figure 7:**
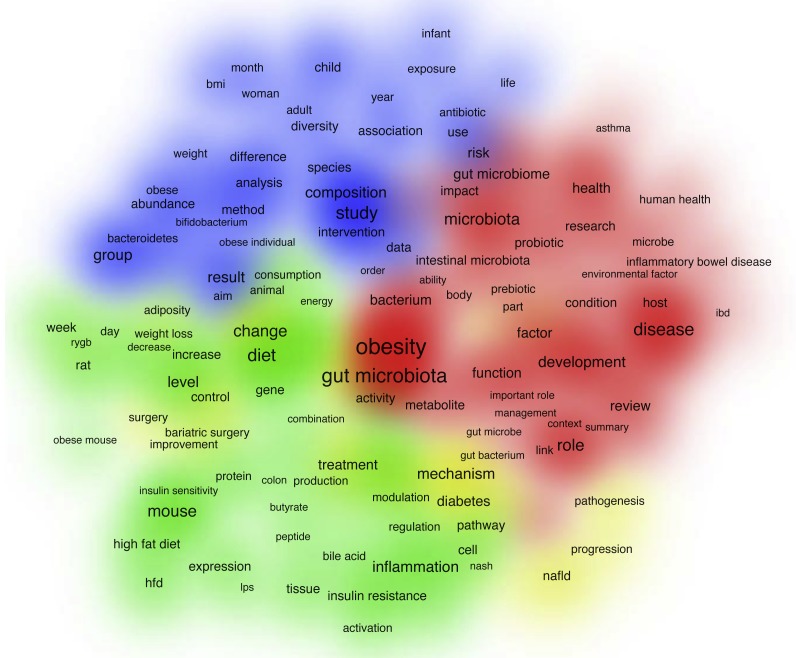
Density map of the most frequently encountered terms extracted from the titles and abstracts of retrieved publications. A number of 195 terms met the threshold with a minimum number of occurrences as 100. The larger circle size or font size indicates higher occurrence.

A density visualization map of most frequently encountered terms is shown in Fig. 7. This analysis was performed based on the terms extracted from the title and abstract fields of retrieved publications; a number of 195 terms met the threshold with a minimum number of occurrences as 100. The term with the highest frequency was obesity (*N* = 4, 677), followed by gut microbiota (*N* = 3, 437), and diet (*N* = 2, 267).

## Discussion

Obesity is a leading cause of preventable death in the US. Obesity induces a number of health problems, both independently and in association with other diseases, such as coronary heart disease, type 2 diabetes, and an increased incidence of several forms of cancer (Feng et al., 2015; Ozturk, 2017; Wang et al., 2015). Some variables such as host genetics, sedentary lifestyle and high-fat diet are identified as etiological factors of obesity (Cheng et al., 2016; Kobyliak et al., 2016), however, the in-depth pathogenesis that accounts for the development of obesity has yet to be disclosed.

Experimentally, model organisms provide an important approach for understanding the cause behind different disorders. Animal models approximate some human diseases, such as diabetes and obesity, and could reproduce many these medical conditions. As is indicated in recent enteric microbiota studies, the community of microorganisms residing in the gastrointestinal tract, is playing a major role in the onset and development of obesity (Duranti et al., 2017; Kvit & Kharchenko, 2017). The roles of microbiota can be explored within the constraints of particular animal model systems, although standard models of inbred mice are limited by their uncontrolled microbiome diversity (Cho & Blaser, 2012).

Intestinal microbiota is involved in the pathogenesis of obesity through various of pathways. Gut microbiota can influence the whole-body metabolism by affecting the host’s energy homeostasis through the mechanism of adjusting the quantity of effector molecules to finally regulate the fat storage in adipocytes (Bai, Zhu & Dong, 2016; De Clercq et al., 2016). Besides, gut saccharolytic microorganisms can degrade complex dietary glycans, which humans cannot utilize directly, and then provide the host with a variety of metabolites. This process may exert extensive influence on the glucose, cholesterol, and lipid metabolism (Duranti et al., 2017).

Modern lifestyles that change the selection pressures on microbiomes could alter exposures to bacteria during the early lives of hosts and thus may contribute to the development of obesity. Antibiotic use in human infancy was significantly associated with obesity development (Ajslev et al., 2011). Alterations in the enteric microbiome also occur when interventions are used to treat obesity (Li et al., 2011). Thus, modulation of the host’s enteric microbiota is a promising way to reduce human obesity (Li et al., 2017b; Zhang et al., 2014).

Bibliometric study is based on the comprehensive analysis of publications’ bibliographic data, such as authors’ affiliations, publication types, source countries, funding and citation information (Pritchard, 1969). In this study, the significance of intestinal microbiota in the progression and management of obesity has been analyzed using a bibliometric approach. The present study is the first time that a bibliometric overview of academic publications on the topic of intestinal microbiota and obesity has been presented. The percentage of intestinal microbiota related publications in the field of obesity research has been soaring in recent years.

North America and Europe are the most prolific areas in this field; the US and China are the top two most productive countries. Several factors are revealed to be significantly associated with the publication numbers of this field. The most positively related factor is GDP, which can partly explain the highest productivity of the US. The HDI and PN also show a positive correlation with the publication numbers, but are weaker than GDP.

The most productive journals in this field are *PLOS ONE*, *Scientific Reports,* and *British Journal of Nutrition*. The most productive institutions are *University College Cork* in Ireland, *Inserm* in France, and *Universite Catholique de Louvain* in Belgium. International collaboration analysis also shows that most of the collaborations are between developed countries. However, a multitude of developing countries are also suffering from the increasing prevalence of obesity (Nasreddine et al., 2017), so this study reveals the necessity of developed countries to support the developing regions in gut microbiota and obesity-related research in order to address this issue on a global scale.

## Conclusions

Intestinal microbiota is playing a significant role in obesity research. Data obtained from this study represent the global research trends, collaboration patterns, and spatial density of the role of intestinal microbiota in obesity. These data are helpful for scientific researchers and public health policymakers in research planning and decision-making of this domain. The current study can also help scientists to locate research hot spots and gaps by offering comprehensive analyses and structured information on this topic.

## Abbreviations

SCR: standard competition ranking
IF: impact factor

##  Supplemental Information

10.7717/peerj.5091/supp-1 Figure S1 The most prolific authors and countries/ regions (A) Top 20 most prolific authors. (B) Top 20 authors with the highest citation numbers per publication. Top 20 most prolific countries/regions according to publication numbers (C) and publication productivity scores (D). (E) The percentages of publications in six continents.

10.7717/peerj.5091/supp-2 Figure S2 Co-authorship analysis The minimum number of documents of an author was 10, 85 meet this threshold. For each of the 85 authors, the total strength of the co-authorship links with other authors was calculated. Thicker lines indicate stronger collaborations. Authors represented with larger circle size or font size had relatively more publications.

10.7717/peerj.5091/supp-3 Figure S3 Density visualization of citation analysis for countries with a minimum productivity of 5 documents, a total of 48 countries and regions meet the threshold

10.7717/peerj.5091/supp-4 Figure S4 International collaboration analysis based on 48 countries and regions which published at least 5 articles on gut microbiota and obesity Thicker lines indicate stronger collaborations. Countries represented with larger circle size or font size had relatively more publications. The United States, Italy and Spain had the most collaborations with other countries.

10.7717/peerj.5091/supp-5 Figure S5 Citation analysis of active organizations A number of 79 organizations met the threshold of at least 1000 citation numbers. Larger circle size indicated more citations.

10.7717/peerj.5091/supp-6 Data S1 Raw data

## Additional Information and Declarations

### Data Availability

The following information was supplied regarding data availability: The raw data has been provided as a Supplemental File.
