# Supplementary figures and images for: Bibliometric analysis of research on the role of intestinal microbiota in obesity
Source: PeerJ. 2018 Jun 29;6:e5091. doi: 10.7717/peerj.5091 (PMC6027659; doi:10.7717/peerj.5091)

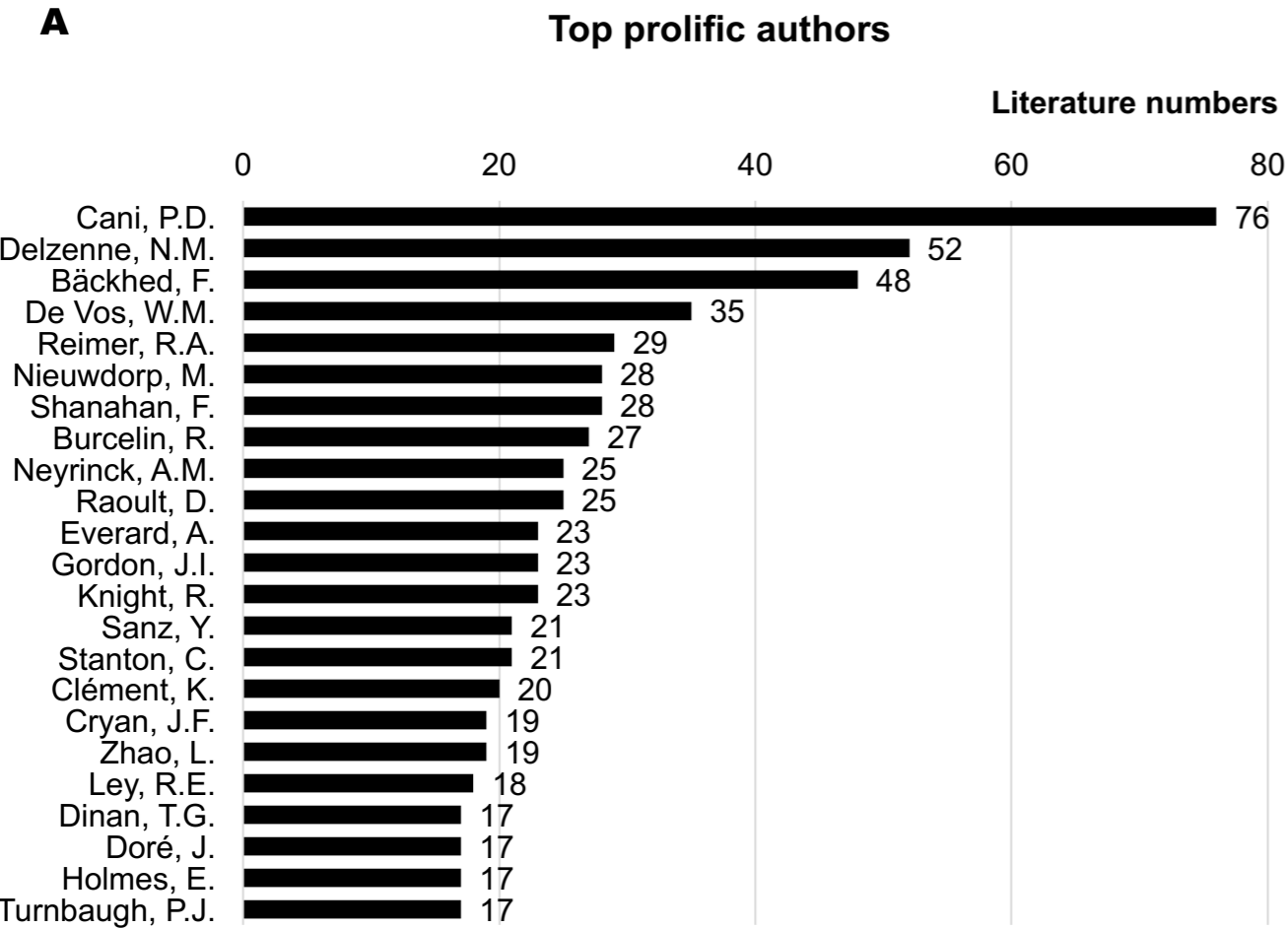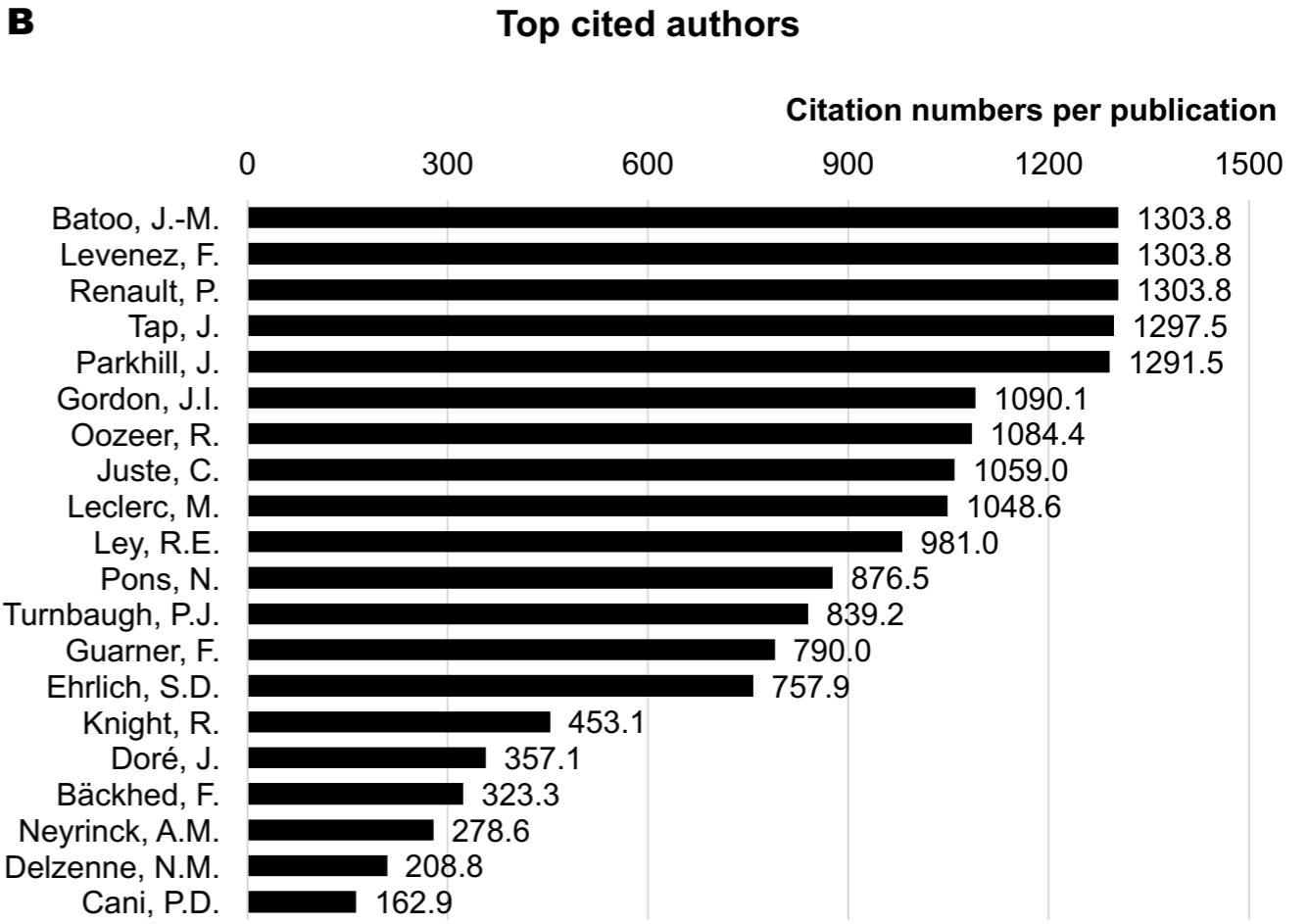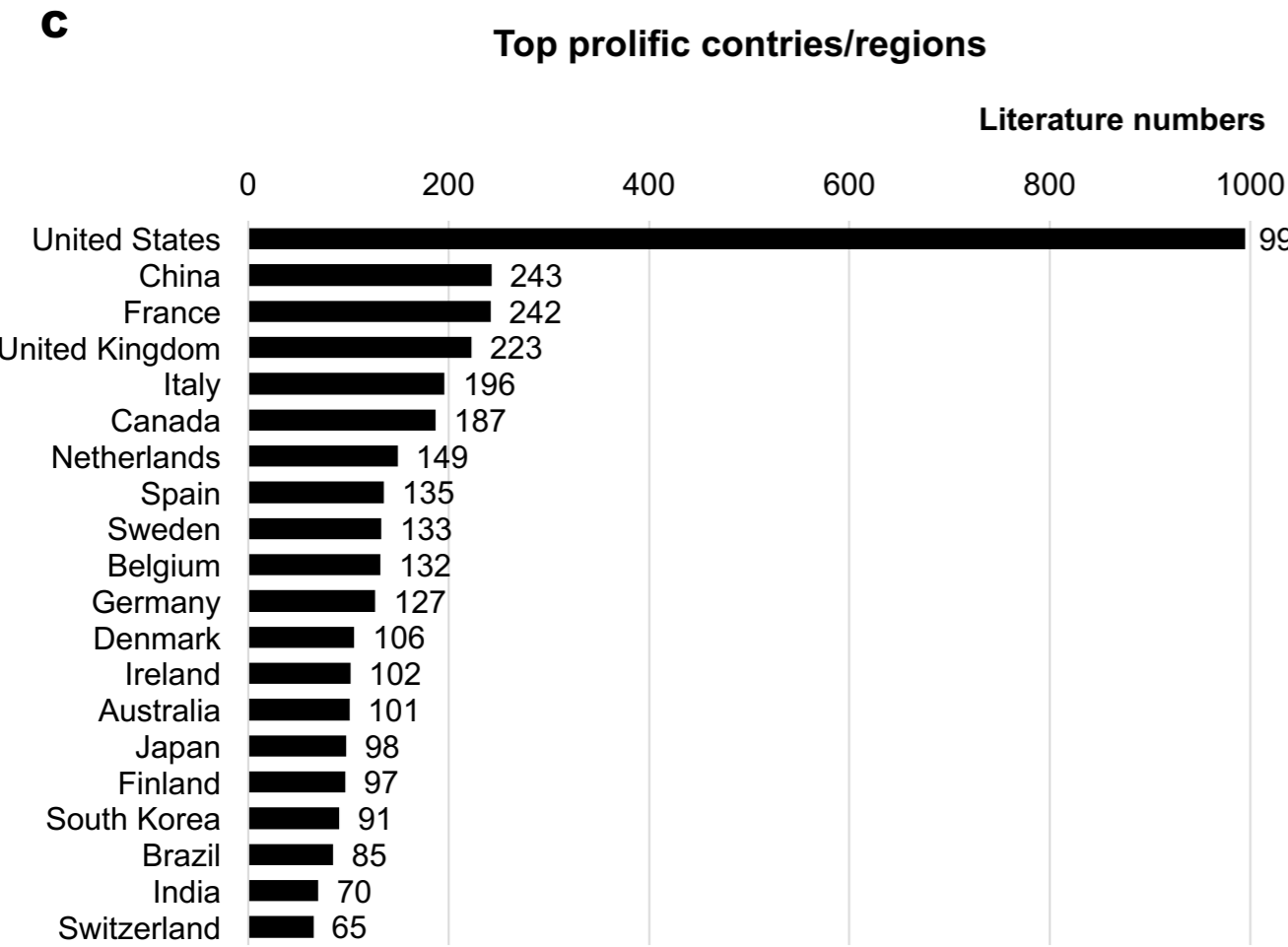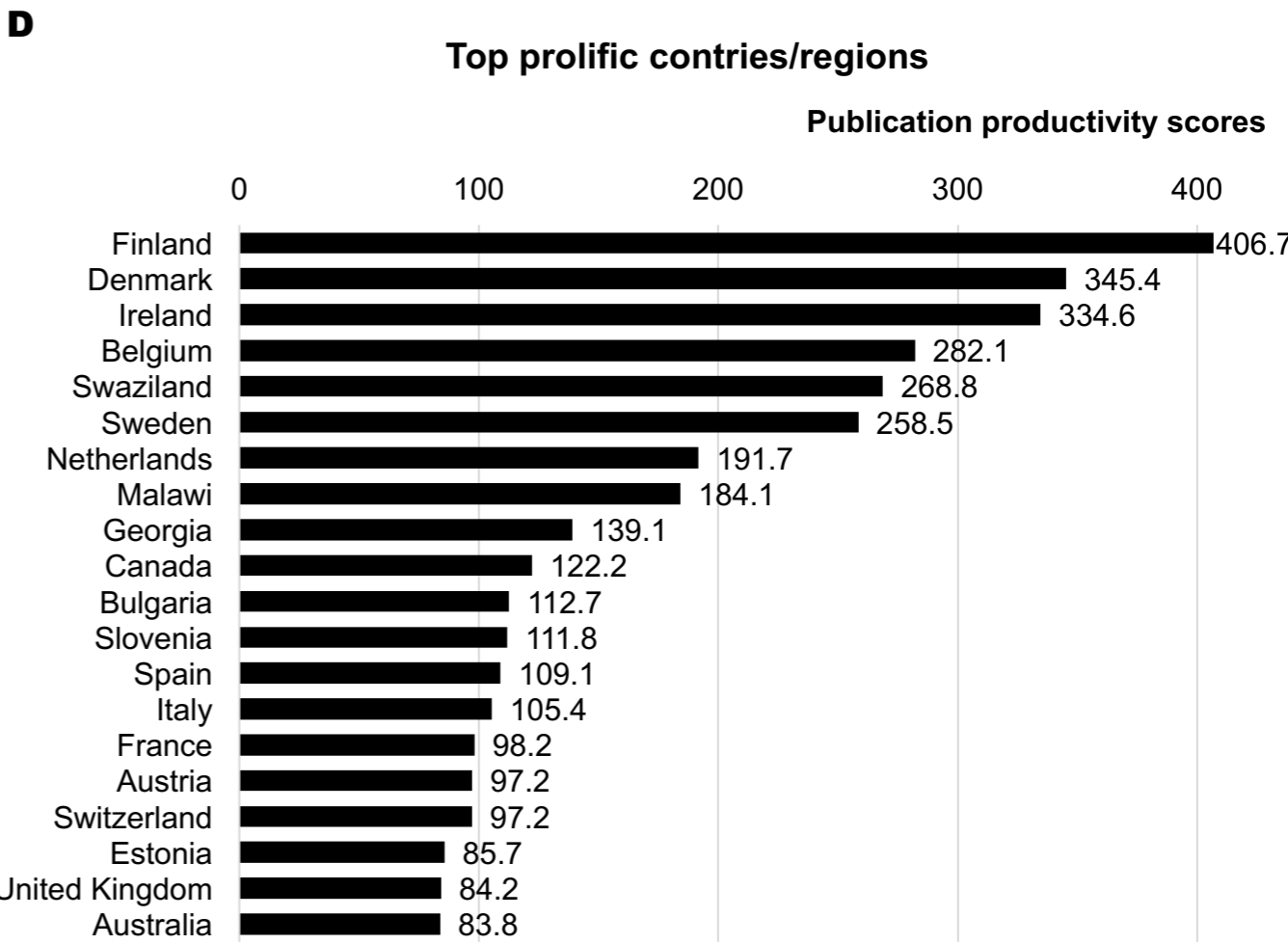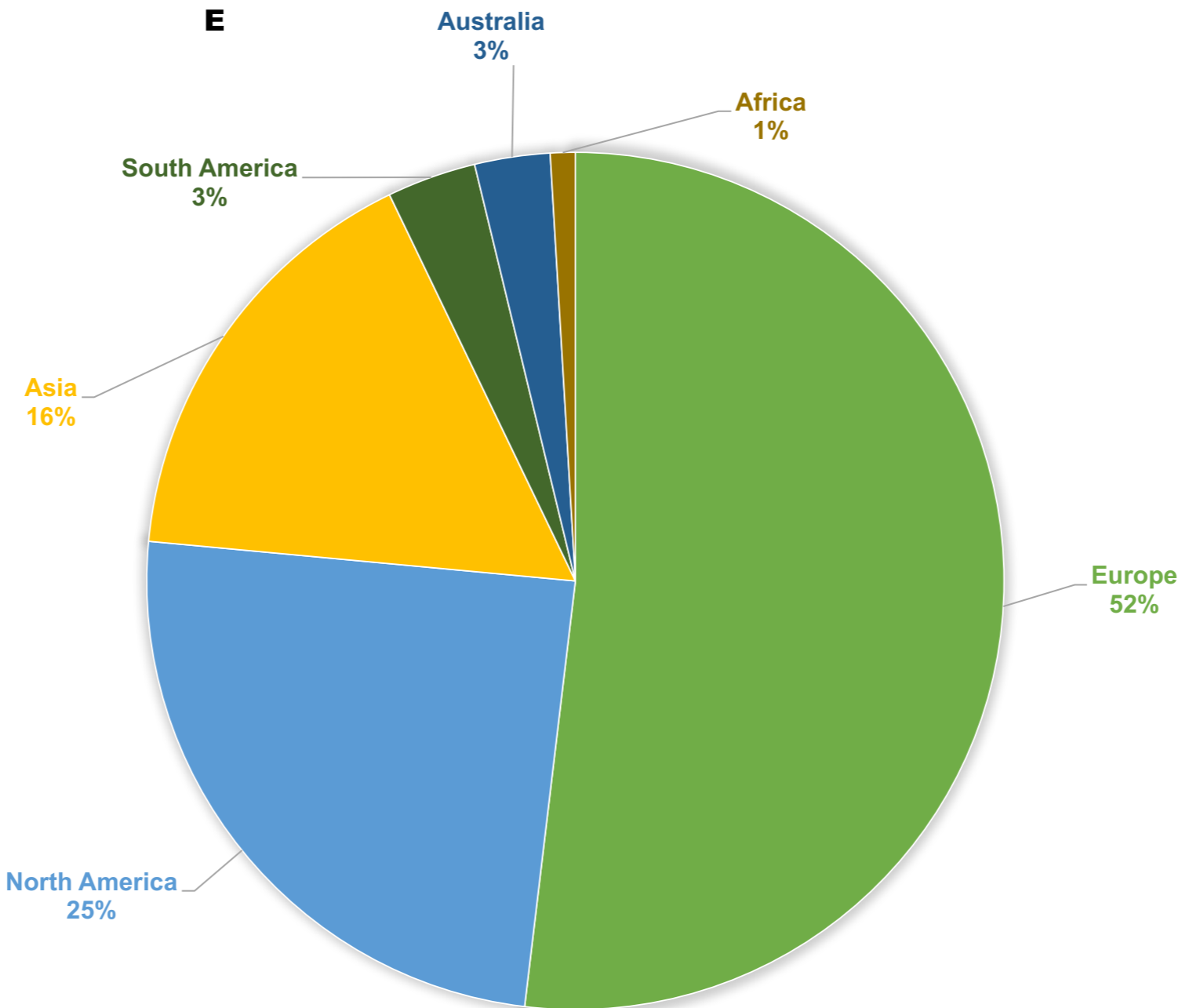

Supplement: Figure S1 — (A) Top 20 most prolific authors. (B) Top 20 authors with the highest citation numbers per publication. Top 20 most prolific countries/regions according to publication numbers (C) and publication productivity scores (D). (E) The percentages of publications in six continents. [file peerj-06-5091-s001.pdf]

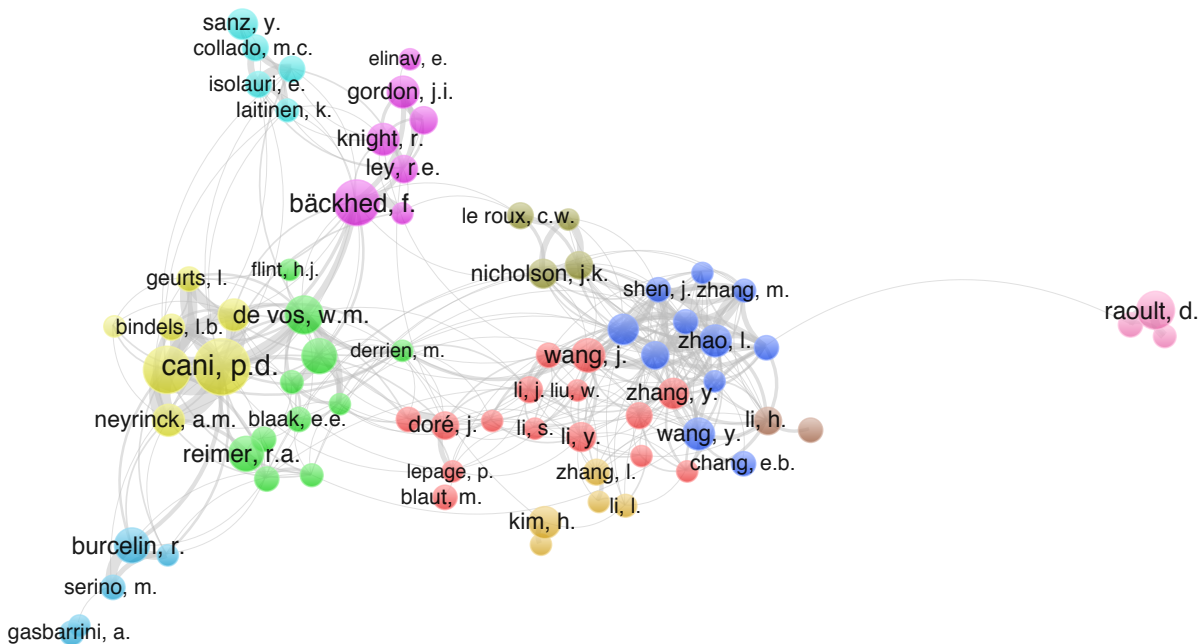

Supplement: Figure S2 — The minimum number of documents of an author was 10, 85 meet this threshold. For each of the 85 authors, the total strength of the co-authorship links with other authors was calculated. Thicker lines indicate stronger collaborations. Authors represented with larger circle size or font size had relatively more publications. [file peerj-06-5091-s002.pdf]

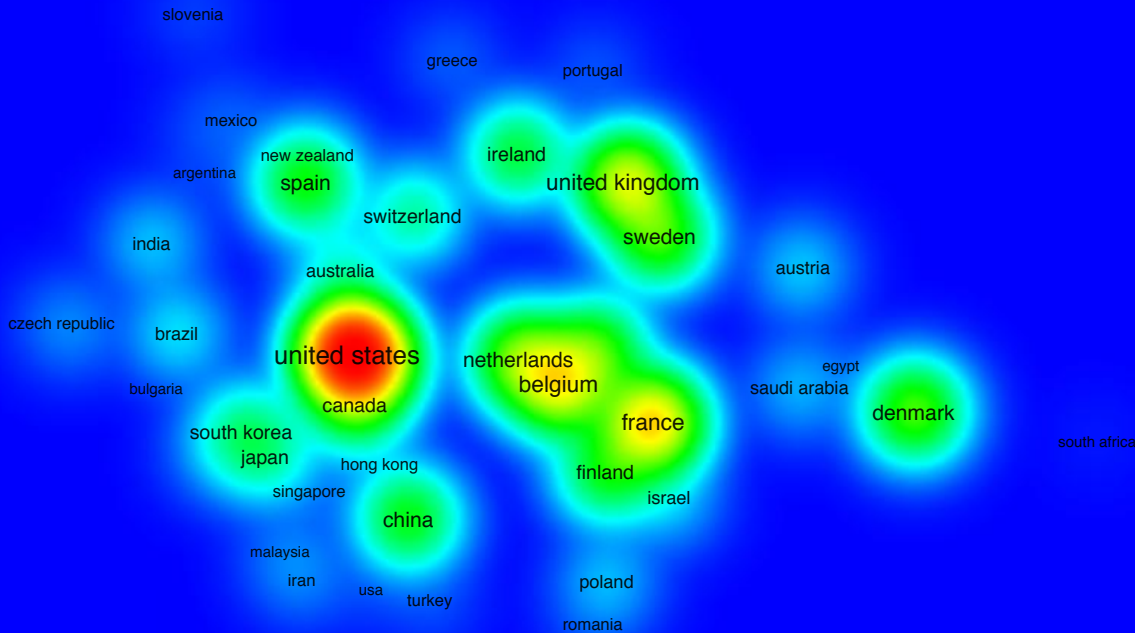

Supplement: Figure S3 [file peerj-06-5091-s003.pdf]

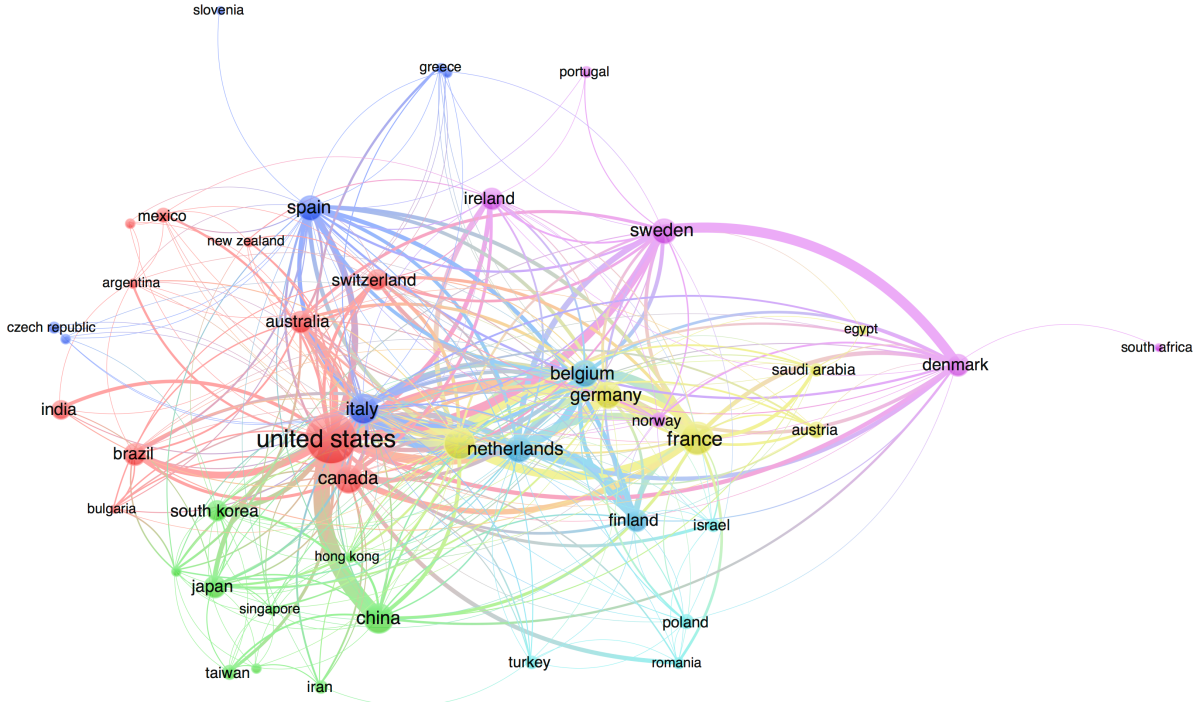

Supplement: Figure S4 — Thicker lines indicate stronger collaborations. Countries represented with larger circle size or font size had relatively more publications. The United States, Italy and Spain had the most collaborations with other countries. [file peerj-06-5091-s004.pdf]

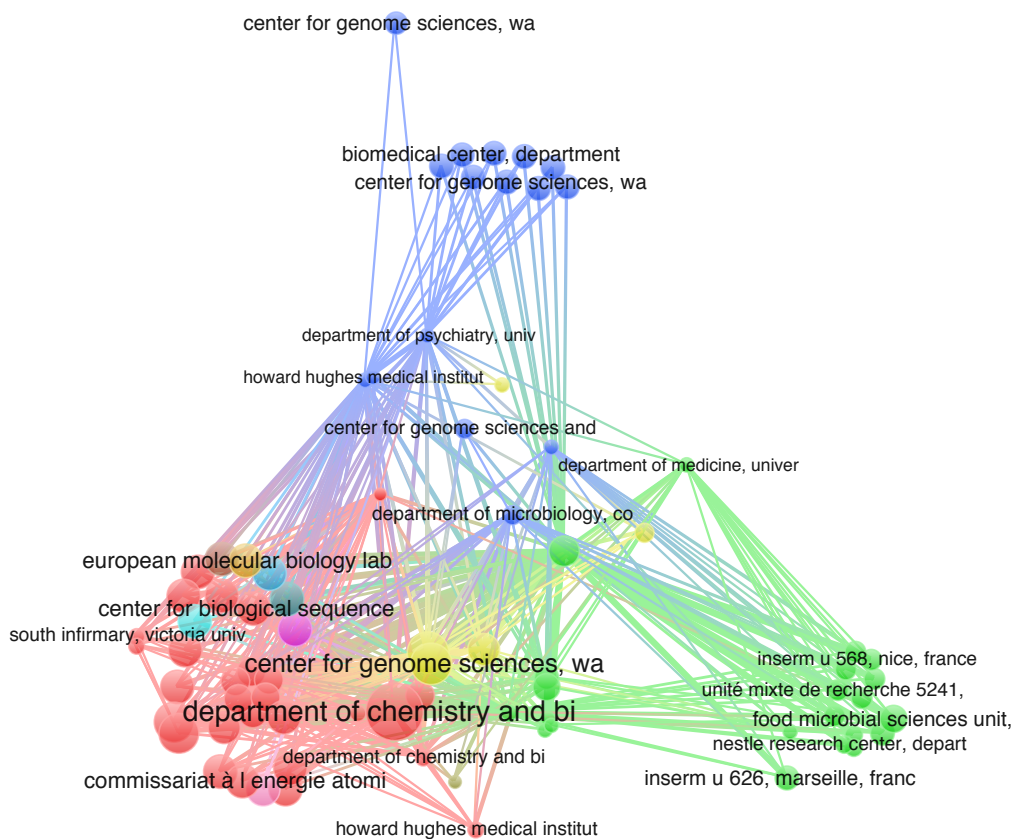

Supplement: Figure S5 — A number of 79 organizations met the threshold of at least 1000 citation numbers. Larger circle size indicated more citations. [file peerj-06-5091-s005.pdf]
